# KEA3: improved kinase enrichment analysis via data integration

**DOI:** 10.1093/nar/gkab359

**Published:** 2021-05-21

**Authors:** Maxim V Kuleshov, Zhuorui Xie, Alexandra B K London, Janice Yang, John Erol Evangelista, Alexander Lachmann, Ingrid Shu, Denis Torre, Avi Ma’ayan

**Affiliations:** Department of Pharmacological Sciences, Mount Sinai Center for Bioinformatics, Icahn School of Medicine at Mount Sinai, One Gustave L. Levy Place, Box 1603, New York, NY 10029, USA; Department of Pharmacological Sciences, Mount Sinai Center for Bioinformatics, Icahn School of Medicine at Mount Sinai, One Gustave L. Levy Place, Box 1603, New York, NY 10029, USA; Department of Pharmacological Sciences, Mount Sinai Center for Bioinformatics, Icahn School of Medicine at Mount Sinai, One Gustave L. Levy Place, Box 1603, New York, NY 10029, USA; Department of Pharmacological Sciences, Mount Sinai Center for Bioinformatics, Icahn School of Medicine at Mount Sinai, One Gustave L. Levy Place, Box 1603, New York, NY 10029, USA; Department of Pharmacological Sciences, Mount Sinai Center for Bioinformatics, Icahn School of Medicine at Mount Sinai, One Gustave L. Levy Place, Box 1603, New York, NY 10029, USA; Department of Pharmacological Sciences, Mount Sinai Center for Bioinformatics, Icahn School of Medicine at Mount Sinai, One Gustave L. Levy Place, Box 1603, New York, NY 10029, USA; Department of Pharmacological Sciences, Mount Sinai Center for Bioinformatics, Icahn School of Medicine at Mount Sinai, One Gustave L. Levy Place, Box 1603, New York, NY 10029, USA; Department of Pharmacological Sciences, Mount Sinai Center for Bioinformatics, Icahn School of Medicine at Mount Sinai, One Gustave L. Levy Place, Box 1603, New York, NY 10029, USA; Department of Pharmacological Sciences, Mount Sinai Center for Bioinformatics, Icahn School of Medicine at Mount Sinai, One Gustave L. Levy Place, Box 1603, New York, NY 10029, USA

## Abstract

Phosphoproteomics and proteomics experiments capture a global snapshot of the cellular signaling network, but these methods do not directly measure kinase state. Kinase Enrichment Analysis 3 (KEA3) is a webserver application that infers overrepresentation of upstream kinases whose putative substrates are in a user-inputted list of proteins. KEA3 can be applied to analyze data from phosphoproteomics and proteomics studies to predict the upstream kinases responsible for observed differential phosphorylations. The KEA3 background database contains measured and predicted kinase-substrate interactions (KSI), kinase-protein interactions (KPI), and interactions supported by co-expression and co-occurrence data. To benchmark the performance of KEA3, we examined whether KEA3 can predict the perturbed kinase from single-kinase perturbation followed by gene expression experiments, and phosphoproteomics data collected from kinase-targeting small molecules. We show that integrating KSIs and KPIs across data sources to produce a composite ranking improves the recovery of the expected kinase. The KEA3 webserver is available at https://maayanlab.cloud/kea3.

## Graphical Abstract

**Graphical Abstract:**
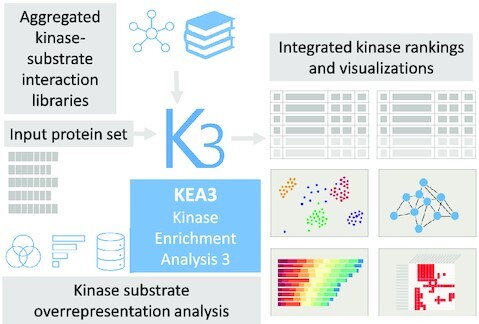
Kinase Enrichment Analysis 3 (KEA3) infers upstream kinases whose putative substrates are overrepresented in a user-inputted list of differentially phosphorylated proteins.

## INTRODUCTION

Protein kinases catalyze the transfer of a phosphate group from ATP to other proteins’ threonine, serine, or tyrosine residues (1). The reversible addition of the phosphate group to a protein can affect the substrate protein activity, stability, localization, and interactions with other molecules (2). Each protein kinase recognizes between one to a few hundred substrates (3). Mass-spectrometry phosphoproteomics experiments can yield over 50,000 unique phospho-peptides that span >75% of all cellular proteins (4). Thus, phosphoproteomics experiments can capture the cellular state of cell-signaling networks. However, kinase activity levels are difficult to discern from the results of such experiments. Since kinases serve a critical and central role in regulating essentially all cellular processes (5), and their aberrant constitutive activation is recognized as a cause of many human cancers (6–10), identifying alterations in kinase state given results from phosphoproteomics experiments is critical.

Protein kinases are one of the most targeted protein families amenable for inhibition by small molecules (11), while most clinically approved protein kinase inhibitors target receptor tyrosine kinases (RTKs) to block cancer proliferation and angiogenesis (12). However, an increasing number of kinase inhibitors for non-oncological indications have been recently approved. New druggable protein kinase targets can be identified by experiments that detect de-regulated kinase-mediated processes contributing to disease progression. For example, mass-spectrometry phosphoproteomics profile the differential phosphorylation states of cellular proteins between two cellular states (13). Such data provides a snapshot of the intracellular signaling networks that are differentially activated between two conditions, for instance, between diseased and healthy cells. The enrichment of known kinase substrates in a set of differentially phosphorylated proteins can serve as a potential marker of the upstream kinases' state and provide insights into physiological and pathophysiological mechanisms (14).

Available tools that predict relevant kinases associated with a set of genes, proteins or phosphorylation sites include Expression2Kinases (X2K) (15,16), PTMsigDB (17), Inference of Kinase Activities from Phosphoproteomics (IKAP) (18), Kinase Perturbation Analysis (KinasePA) (19) and Kinase Substrate Enrichment Analysis (KSEA) (20). X2K is a web tool that predicts cell-signaling pathways upstream from differentially expressed mRNAs. It first performs transcription factor enrichment analysis (TFEA) (21–23); it then connects these factors based on known protein-protein interactions (24), and then performs kinase-enrichment analysis (KEA) to rank the most relevant protein kinases. One of the limitations of X2K is that it performs the enrichment analysis at the protein level. PTMsigDB is a database of post-translational modification site (PTM-site) specific signatures curated from publications, including kinase state signatures. PTM Signature Enrichment Analysis (PTM-SEA) is an R package for modified gene set enrichment analysis (GSEA) used to query a user-inputted set of PTMs against the PTMsigDB database. IKAP consists of a collection of MATLAB functions that estimate kinase state from a phosphoproteomics dataset by minimizing a cost function relating the kinase state to the phosphosite measurements. KinasePA, available as an R package called directPA and as a webserver, uses experimentally determined kinase-phosphosite interactions to perform kinase enrichment analysis applied directly to mass-spectrometry proteomics readouts. KSEA is a web-based tool that uses known kinase-phosphosite relationships to compute a normalized score for each protein kinase based on the relative hyper- or hypo-phosphorylation of its substrates.

In contrast with prior implementations, Kinase Enrichment Analysis 3 (KEA3), which also computes kinase overrepresentation for a query of human or mouse protein or gene sets, integrates kinase-substrate interactions (KSI) from a multitude of resources to compute a composite kinase enrichment score. To develop KEA3, we adapted the web application and benchmarking framework previously deployed for creating the transcription factor enrichment analysis tool ChEA3 (23). To benchmark KEA3, we evaluated the utility of publicly available KSI, PPI, co-occurrence, and co-expression data to compute overrepresentation of putative kinase substrates for a user inputted protein set. KEA3 expands significantly on and is a complete reimplementation of KEA (25). KEA3 contains more kinase-substrate libraries, incorporates three independent systematic benchmarks, and integrates results across data sources to improve recovery of the expected upstream kinases. This integration method performs consistently better than any single library across the three benchmarks. Two of the KEA3 benchmarks also demonstrate the utility of KEA3 for analyzing signatures from drug perturbation experiments to infer candidate kinase targets for kinase inhibitor drugs and small molecules. To further demonstrate the utility of KEA3, as a case study, we applied kinase enrichment analysis to phosphoproteomics data collected from recent SARS-CoV-2 studies.

## MATERIALS AND METHODS

### Arriving at a consensus list of human kinases

We mapped protein and gene symbols to HGNC-approved gene symbols (26) and discarded gene or protein symbols that did not map using synonym or alias matching. To accomplish this, we developed an R package called genesetr (https://github.com/MaayanLab/genesetr). The union of kinome lists from Manning *et al.* (5), Miranda-Saavedra and Barton (27), and the Illuminating the Druggable Genome (IDG) project (11) produced the set of 520 unique KEA3 HGNC-mappable human protein kinases.

### Protein–protein and kinase-substrate interaction libraries

The KEA3 gene-set libraries are kinase-substrate sets aggregated from several resource types: PPI, KSI, co-occurrence, and transcript co-expression. One additional library not described below, termed STRING, was composed of all human kinase-protein links in version 11.0 of the STRING database (28). The code used to generate the KEA3 libraries and for benchmarking KEA3 can be found at https://github.com/MaayanLab/KEA3webData.

The PPI and KSI datasets (Tables 1 and 2) include interactions where at least one interacting partner was a member of the KEA3 consensus kinase set. Within each dataset, all kinases are human kinases that have at least five distinct putative human protein substrates. Kinase-interacting proteins were collected from the following PPI databases: BioGRID (29), mentha (30), hu.MAP (31), prePPI (32,33), MINT (34,35), HIPPIE (36), PIPs (37,38), PSOPIA (39), REACTOME (40), Cheng *et al.* (41) and STRING (28). The BioGRID and MINT databases contain PPIs from high- and low- throughput experiments that were manually curated from the literature. Mentha is a PPIN that contains molecular interactions aggregated and updated weekly from MINT, IntAct (42), BioGRID, MatrixDB (43), and the Database of Interacting Proteins (DIP) (44). HIPPIE aggregates experimentally determined PPIs from IntAct, MINT, BioGRID, HPRD (45), DIP, BIND (46) and MMPI (47). Cheng et al. used PPIs collected from IntAct, MINT, BioGRID, DIP, HPRD and MIPS MPact (48). There are overlaps and redundancy among these databases, especially those that aggregate PPIs from the literature and other PPI databases. We examined each of them individually despite these overlaps because each incorporates different combinations of resources with varying reliability, quality, and coverage.

**Table 1.** PPI databases used to generate the KEA3 kinase-substrate libraries

PPI database	Dataset	Version	KEA3 library name
BioGRID (44)	Multi-validated Physical Interactions	3.5.175	*BioGRID*
mentha (45)	Binary interactions with scores	5 August 2019	*mentha*
hu.MAP (46)	Edge table predictions (prob. > 0.5)	9 August 2019	*Hu.MAP*
prePPI (47)	High-confidence predictions (prob. > 0.5)	10 August 2019	*prePPI*
MINT (48)	*Homo sapiens*	9 August 2019	*MINT*
HIPPIE (49)	Hippie	2.2	*HIPPIE*
PIPs (50,71)	Predicted Interactions with Score ≥1	10 August 2019	*PIPs*
PSOPIA (51)	Dset2_pos_4430	11 August 2019	*PSOPIA*
REACTOME (61)	PPIs derived from REACTOME Pathways	11 August 2019	*REACTOME*
Cheng et al. (52)	PPIN in Supplementary Table S1	static	*Cheng.PPI*
STRING (29)	Interaction types for protein links annotated with ‘Binding’	11	*STRING.bind*

**Table 2.** KSI databases used to generate the KEA3 kinase-substrate libraries

KSI database	Dataset	Version	KEA3 library name
PhosphoSitePlus (34)	*Kinase-substrate*	30 July 2019	*PhosphoSitePlus*
PhosD (65)	Predictions resulting from model trained on Phospho.ELM	5 August 2019	*PhosD.ELM*
PhosD (65)	Predictions resulting from model trained on PhosphoSitePlus	9 August 2019	*PhosD.PSP*
PhosD (65)	Predictions resulting from model trained on all KSINs	10 August 2019	*PhosD.All*
PhosphoNetworks (63)	rawKSI	9 August 2019	*PhosphoNetworks.rawKSI*
PhosphoNetworks (63)	comKSI	9 August 2019	*PhosphoNetworks.comKSI*
PhosphoNetworks (63)	refKSI	9 August 2019	*PhosphoNetworks.refKSI*
PTMsigDB (16)	Kinase signature subset of the Uniprot human dataset	1.9.0	*PTMsigDB*
Cheng et al. (52)	KSIN in Supplementary Table S1	Static	*Cheng.KSI*
Phospho.ELM (64)	Vertebrate database dump	9.0	*Phospho.ELM*

Reactome (49) is a manually curated and peer-reviewed pathway database with annotations that generally focus on the most extensively studied pathways and molecules. hu.MAP (31) integrates thousands of published mass spectrometry (MS) experiments to find all interactions not identified in the original publications. We also constructed a library from the experimentally derived datasets used for testing the PSOPIA PPI prediction model. PIPs and prePPI both consist of predicted PPIs. PIPs (37) is using a naïve Bayes classifier that integrates information from expression, orthologs, domain co-occurrence, PTMs, and subcellular localization. PrePPI (32) also uses a Bayesian framework but relies on three-dimensional structural information in addition to functional, evolutionary, and expression information to make PPI predictions.

Putative KSIs were collected from PhosphoNetworks (50), Phospho.ELM (51), PTMsigDB (17), PhosphoSitePlus (52), PhosD (53) and Cheng *et al.* (41). PhosphoNetworks relies on a combined protein microarray and computational strategy to construct human phosphorylation networks (54,55). PhosphoNetworks ‘raw’ KSIs consist of kinase-substrate relationships (KSRs) identified by protein microarray. PhosphoNetworks ‘reference’ KSIs consist of high-confidence KSIs that were filtered by multiple criteria and validated by transfection experiments. Finally, the PhosphoNetworks ‘combination’ KSIs consist of the union of the reference KSIs and KSIs that were manually curated from the literature. Phospho.ELM is a database of experimentally verified protein phosphorylation sites in eukaryotes, annotated with the phosphorylating kinase when known. PTMsigDB is a collection of phosphosite signatures of kinase activities, perturbations, and signaling pathways curated from the literature. PhosphoSitePlus is a database of manually curated kinase-substrate interactions from thousands of publications. PhosD predicts kinase-substrate interactions based on protein domains. We examined three PhosD kinase libraries generated by the PhosD model trained on Phospho.ELM data, on PhosphoSitePlus data, and on multiple datasets. Cheng *et al.* (41) constructed a KSIN from Phospho.ELM, HPRD, PhosphoNetworks, and PhosphoSitePlus. Finally, the STRING resource is a collection of direct and indirect protein-protein interactions (28). To generate the STRING.bind library, we subset the STRING interactions to only those interactions that were annotated as involving physical binding to generate the STRING.bind library. We also used the entire STRING database to form the STRING library, including physical interaction, co-expression, co-occurrence in the literature, and evolutionary co-occurrence, among other association types.

### Gene co-expression libraries

To create the KEA3 gene co-expression libraries, all GTEx RNA-seq samples were downloaded from the GTEx web server. Samples were quantile-normalized, and for duplicate genes, only the genes with the most significant variance were retained. For each kinase, the 300 genes with the most significant Pearson's correlation coefficients were selected to generate the kinase sets in the *GTEx.coexp* library. To create the *ARCHS4.coexp* library, human RNA-seq samples were downloaded from ARCHS4 (56). Fifty-thousand samples were randomly selected for co-expression analysis and then processed in the same way as for the GTEx data to generate the *ARCHS4.coexp* library.

### Generating the benchmarking datasets

The Characteristic Direction (CD) method (57) was used to compute gene expression signatures from 329 kinase perturbation experiments containing 96 kinases. The list of studies was obtained from the manually curated signatures in the CREEDS resource (58). The perturbations include knockdowns, knockouts, overexpression, constitutively active mutants, chemical activation, and chemical inhibition of single kinases followed by microarray profiling. Gene sets containing the top 600 differentially expressed genes were determined by the absolute value of the Characteristic Direction coefficients constructed for each perturbation experiment. We term this benchmarking dataset *KinCREEDSupdn*.

For the *DrugL1000updn* benchmarking dataset, LINCS L1000 drug perturbation CD signatures retrieved via the L1000FWD API were subset to drugs with known kinase targets using the L1000FWD drug target annotations (59). For each LINCS perturbation identifier, the signature with the greatest cosine similarity from the batch center was selected. The union of the most significant upregulated and downregulated genes was used to compose 292 signatures.

The human PTMsigDB (17) phosphoproteomics signatures were derived from PhosphoSitePlus (52) quantitative mass-spectrometry experiments. Entries were subset to drug perturbations with known kinase targets. Drug targets were obtained from L1000FWD drug annotations (59). Drug perturbations with fewer than five associated HGNC-mappable proteins were discarded, resulting in a benchmarking set of 15 phosphoproteomics drug perturbation signatures which cover 15 unique drugs with 98 annotated kinase targets, 50 of which are unique. We term this set *PTMsigDB.drug*.

### Assessing inter-library concordance

The Fisher's Exact Test (FET) was used to compute similarity between all pairs of protein sets of the 24 kinase-substrate libraries with a default background of 20,000 genes. For a library pair A and B, an integer ranking of the protein sets in B, termed the ‘prediction library’ was produced for each protein set in A, termed the ‘query library,’ based on the FET *P*-values. A rank of 1 represented the most significant FET *P*-value and a rank of *k* represented the least significant FET *P*-value where *k* is the number of protein-sets in the ‘prediction library’. The rankings were then scaled from 1/*k* to 1. An empirical cumulative distribution function (ECDF) was computed from the scaled ranks of the protein set pairs in A and B that represent the same kinase. The ranks were scaled to values between 0 and 1 to obtain an area under the ECDF (AUECDF) for each library pair AB and BA.

### Benchmarking libraries and integration methods

Each protein set from the *KinCREEDSupdn*, *DrugL1000updn* and *PTMsigDB.drug* benchmarking datasets was submitted to KEA3 and benchmarked. Kinases were ranked within each library according to the FET *P*-values, with ties broken randomly. Ranks within each library were then scaled between 0 and 1. The R package PRROC was used to compute the area under the receiver-operating characteristic (AUROC) curve and Precision-Recall (PR) curve for each library. The positive class consists of the scaled ranks of the ‘true’ kinase(s) associated with the query protein set. The negative class consists of the scaled ranks of all other kinases that were not associated with the query set. To generate PR curves, we downsampled the negative class to the same size as the positive class, similarly to the method described by Garcia-Alonso *et al.* (60). Each library has a different number of kinases and therefore has a different ‘random classifier’ PR curve. By down sampling the negative class to the same size as the positive class, a random classifier would have a PR area under the curve (AUC) of 0.5. PR curves were bootstrapped in this manner 1000 times and then the mean PR AUC was reported. The base R function *approx()* was used to interpolate between all points from the 1000 PR curves in order to generate composite PR curves for each library and integration method for visualization. We also employed an additional measure of performance by letting r be the scaled ranks of the ‘true’ kinase(s) associated with the protein set queries. We then examined the ECDF of this set of ranks, *D*(*r*). If the ‘true’ kinases do not display preferentially low or high ranks, then we expect a uniform distribution *D*(*r*) = *U*. We examined *D*(*r*) – *U* for significant deviations from zero to evaluate different libraries and methods. Anderson-Darling tests implemented via the *goftest* R package were used to evaluate the null hypothesis, *D*(*r*) = *U*.

### Kinase enrichment analysis

KEA3 uses Fisher's Exact Tests with a background set of 20,000 genes to compute the significance of the overlap between the query input protein set and each protein set in the KEA3 protein set libraries. An integer rank from 1 to *k* for each protein set in a library size of *k* indicates sets with the lowest and highest *P*-values accordingly. A scaled rank is computed by dividing each integer rank by *k*. Thus, for a single query, there is one kinase rank list for each protein set library in KEA3. False discovery rates (FDRs) are computed via the Benjamini-Hochberg correction for each library separately. Out of the 24 candidate libraries, rank lists for the 11 final KEA3 libraries which met the benchmarking threshold are integrated via the MeanRank and TopRank methods (23). MeanRank is calculated from re-ranking a composite list of kinases by each kinase's mean integer rank across all libraries containing that kinase. A composite list of kinases is re-ranked with each kinase's best-scaled rank across all libraries to calculate TopRank.

### The KEA3 web application

The backend of KEA3 is a Java servlet running on Tomcat 9 (61). The user interface was constructed with jQuery, Bootstrap and the web template application Mobirise (62). The web application runs in a Docker (63) container. The KEA3 source code repository is available at https://github.com/maayanlab/KEA3web.

### Kinase co-expression network visualization

Weighted Gene Co-expression Network Analysis (WGCNA) (64) was applied to GTEx (65), ARCHS4 (56) and TCGA expression data to generate interactive views of the human kinome regulatory network. Prior to applying WGCNA on these datasets, these large-scale collections of gene expression datasets were quantile-normalized and filtered to include only protein kinases. WGCNA with default parameters was then applied to subsets of each dataset separately: the GTEx gene expression dataset; 100 random RNA-seq samples for each of 18 tissue types pulled from the ARCHS4 database; and 100 samples from each of 96 cancer types in the TCGA expression dataset. The three resulting networks were clustered using Cytoscape (66) with the Allegro Fruchterman-Reingold Force Directed Layout plugin, and then visualized on the KEA3 results page using D3.js (67). To annotate the GTEx, ARCHS4 and TCGA networks, WGCNA module eigengenes were correlated to GTEx tissue sample labels, ARCHS4 sample labels, and TCGA tumor types, respectively. Nodes were colored by the most significant tissue/tumor correlation to their parent module.

### Kinase co-regulatory network visualization

A kinase co-regulatory network was constructed from all kinase-kinase interactions described by the 11 KEA3 libraries. Edges are directed where kinase-substrate evidence supports the interaction and are undirected in the case of PPI or unspecified interaction evidence only. The network is a subset based on the top kinase results from a user query and is visualized using D3.js.

## RESULTS

### Computing kinase enrichment

KEA3 computes kinase substrate overrepresentation for a query protein set against 11 kinase-substrate set libraries covering 520 unique protein kinases (Table 3). KEA3 uses the FET to compare a user-submitted protein set query to each kinase-substrate set in each KEA3 library. A kinase ranking is returned for each library separately based on the FET *P*-values. For a given library, kinase rankings range from 1, which corresponds to the most significant FET, to *k*, where *k* is the number of kinase-substrate sets in the library. KEA3 results also return a scaled rank from 1/*k* to 1.

**Table 3.** Summary of the KEA3 libraries. Dark kinases are determined based on a list published by the Illuminating the Druggable Genome Project (28)

	Library	Unique kinases	Dark kinases	Unique set members	Mean set size	Included in KEA3 tool
Kinase-Substrate Libraries	*PhosphoSitePlus*	165	8	2269	32	N
	*PhosD.ELM*	161	10	3799	127	N
	*PhosD.PSP*	212	23	5565	16	N
	*PhosD.All*	339	66	6544	66	Y
	*PhosphoNetworks.rawKSI*	285	77	1914	83	N
	*PhosphoNetworks.comKSI*	181	33	1115	23	N
	*PhosphoNetworks.refKSI*	164	32	717	21	N
	*PTMsigDB*	163	8	2262	32	Y
	*Cheng.KSI*	227	35	2154	31	Y
	*Phospho.ELM*	39	0	418	16	N
Protein-protein Interaction Libraries	*BioGRID*	240	31	2251	24	Y
	*mentha*	474	124	8639	72	Y
	*Hu.MAP*	33	10	294	12	N
	*prePPI*	519	149	14 382	658	Y
	*MINT*	156	9	1383	72	Y
	*HIPPIE*	474	127	8798	97	Y
	*PIPs*	266	41	2068	50	N
	*PSOPIA*	44	2	493	14	N
	*REACTOME*	178	10	1209	22	N
	*Cheng.PPI*	376	73	4678	40	Y
	*STRING.bind*	432	99	5254	72	Y
Kinase Co-expression Libraries	*ARCHS4.coexp*	515	148	16 711	300	N
	*GTEx.coexp*	515	148	17 769	300	N
Other	*STRING*	514	148	18 213	1235	Y

### Constructing the KEA3 libraries

**Figure 1.**
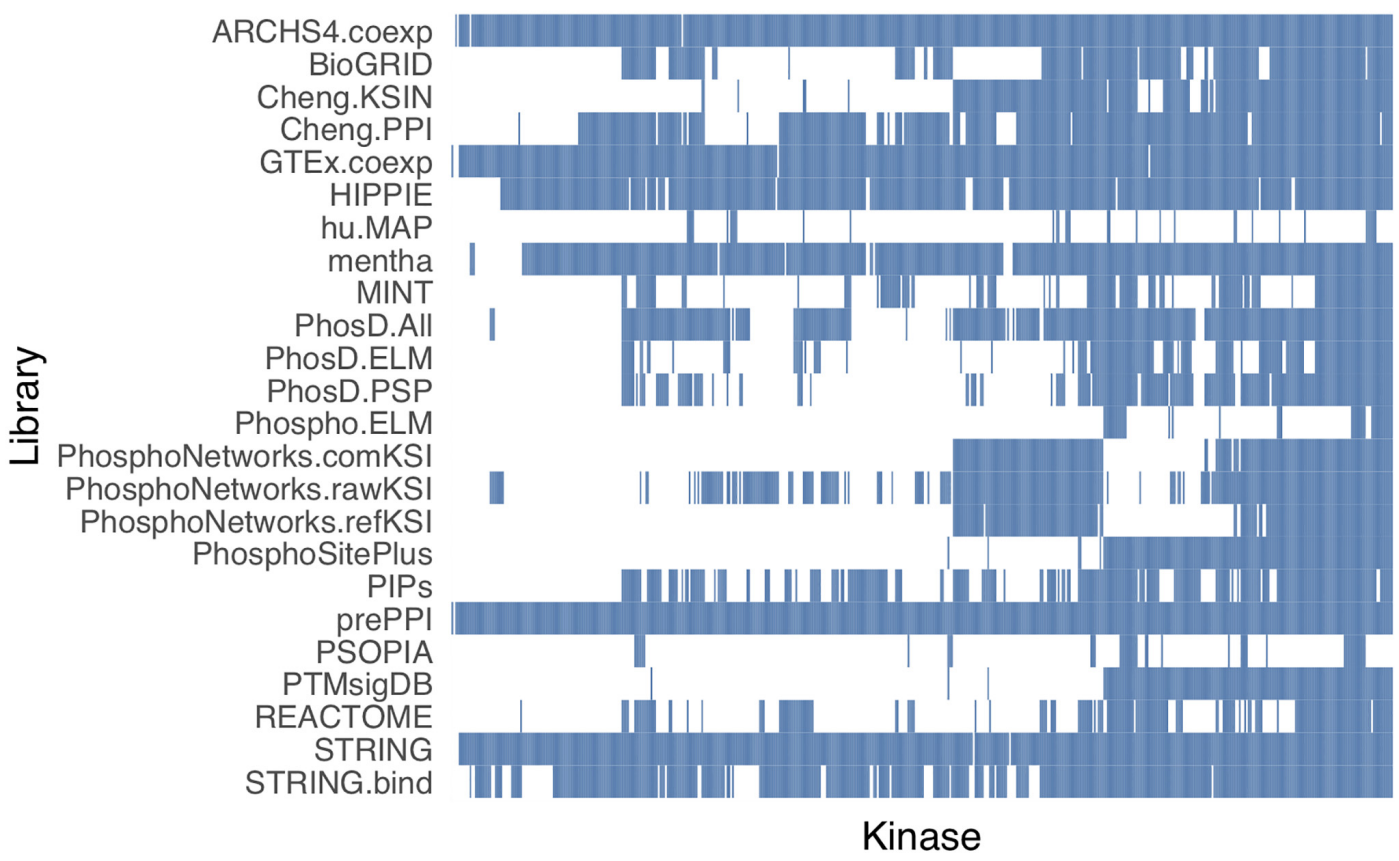
Heatmap representing the kinase coverage of the KEA3 libraries.

We constructed 24 known and putative kinase-substrate libraries with publicly available data from co-expression analysis, experimentally measured PPIs, predicted PPIs, measured KSIs, predicted KSIs and a database that integrates the interactions mentioned above with literature associations and evolutionary associations (28). Each resource was subset to interactions and associations involving the 520 HGNC-mappable protein kinases identified in Manning *et al.* (5), Miranda-Saavedra and Barton (27), and the Illuminating the Druggable Genome (IDG) project (11) (Table 3, Figure 1). We evaluated the 24 candidate libraries with three benchmarking datasets. We also assessed the performance of KEA3 in recovering perturbed kinases from microarray kinase gene perturbation experiments, kinase drug targets from microarray drug perturbation experiments, and kinase drug targets from phosphoproteomics drug perturbation experiments. We then selected the top 11 libraries for use in the KEA3 webserver based on these benchmarking results. We also benchmarked two methods that integrate the results from each of the 11 selected top KEA3 libraries to generate a composite kinase ranking.

### Assessing inter-library predictability

**Figure 2.**
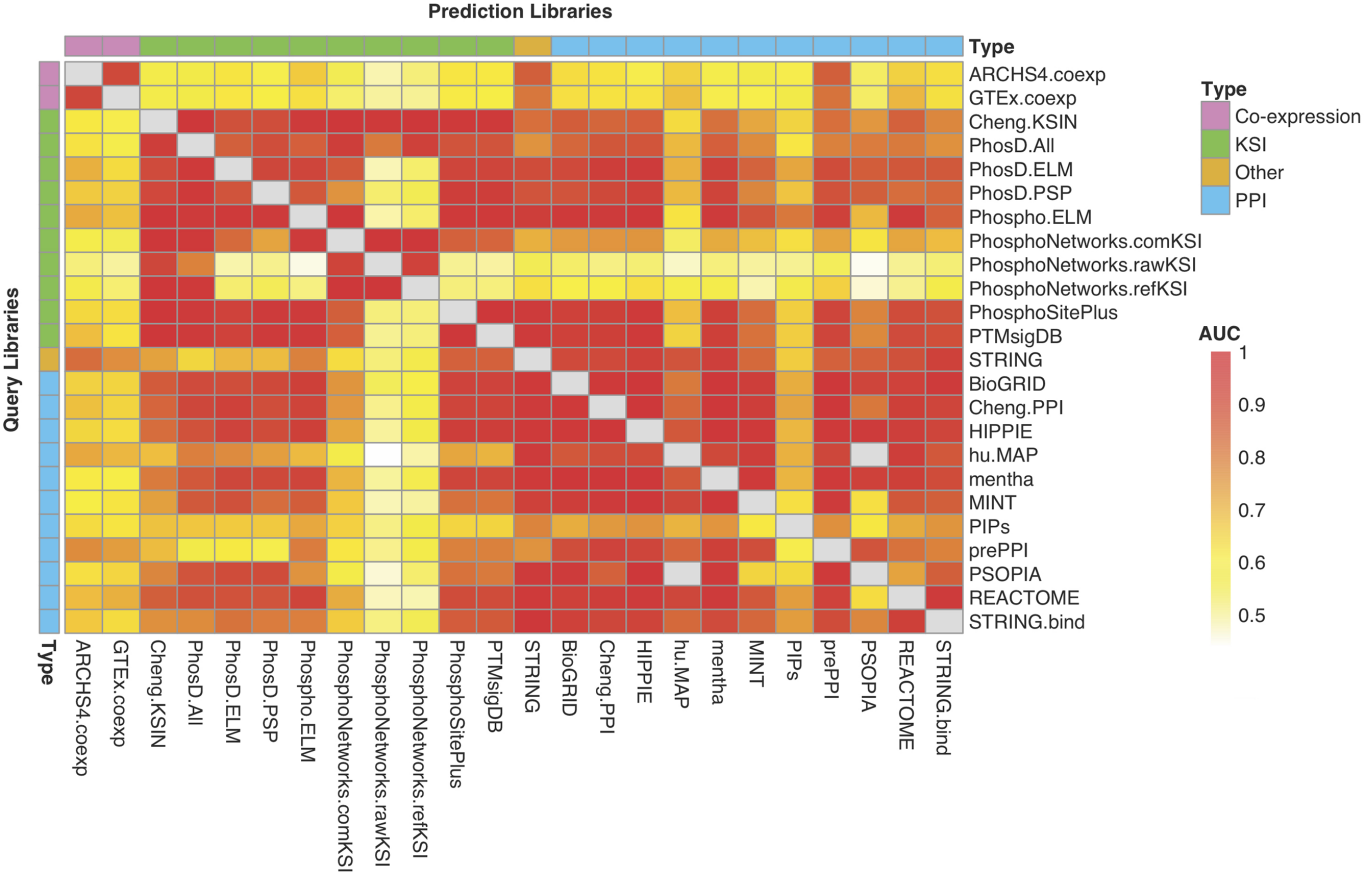
Heatmap showing all pairwise library comparisons. The tile color shows the AUC of the ECDF that represents how well a given ‘prediction’ library was able to recover the ‘correct’ kinase associated with gene sets from the ‘query’ library.

We examined all pairs of the candidate KEA3 libraries, where one library was designated as the ‘query’ library, and the other library was designated as the ‘prediction’ library. We ranked all the protein sets of the prediction library according to the *P*-values resulting from pairwise FETs calculated for each kinase-associated gene set in the query library. We then constructed empirical cumulative distribution functions (ECDFs) from the scaled rank values where the two sets being compared were associated with the same kinase. The areas under the ECDFs (AUECDFs) were evaluated to visualize pairwise library predictability (Figure 2).

### Benchmarking the KEA3 libraries

We used three independent benchmarking datasets to evaluate the initial 24 KEA3 libraries. Each benchmarking dataset consists of gene/protein sets that are each associated with one or more kinases. The *KinCREEDSupdn* benchmark dataset consists of gene sets extracted from 329 kinase loss-of-function/gain-of-function (LOF/GOF) human and mouse microarray experiments mined from GEO by contributors to a crowd-sourcing project (58). Each gene set within the *KinCREEDSupdn* dataset consists of the 600 most differentially regulated genes from each kinase perturbation experiment. The *DrugL1000updn* is comprised of statistically significant up-regulated and down-regulated genes extracted from transcriptome-wide signatures imputed from the LINCS L1000 drug perturbation signatures (59). We took the subset of the drug perturbation signatures that have annotated kinase drug targets, such that each of the 292 *DrugL1000updn* gene sets is associated with one or more protein kinase drug targets. The third benchmarking set, *PTMsigDB.drug*, consists of human phosphoproteomic drug perturbation signatures derived from quantitative MS studies that measured differential phosphorylation states before and after drug perturbations (17,52). The 15 *PTMsigDB.drug* signatures represent 15 unique drugs with 98 annotated kinase targets total, 50 of which are unique kinase targets.

**Figure 3.**
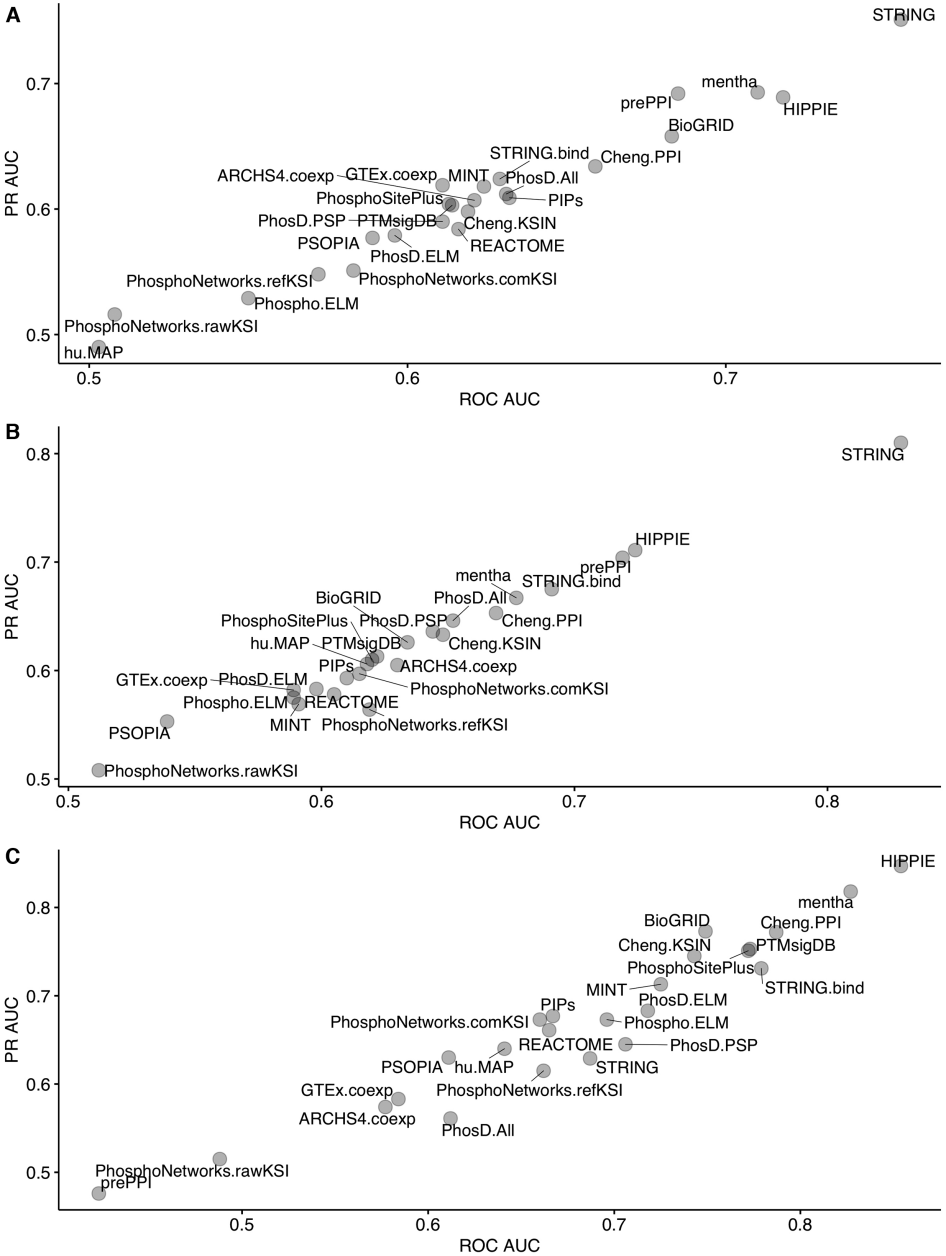
ROC AUC and mean PR AUC over 1000 bootstrapped PR curves for all candidate KEA3 libraries for recovering the perturbed kinases and kinase drug targets from three benchmarking datasets. (**A**) *KinCREEDSupdn*; (**B**) *DrugL1000updn;* (**C**) *PTMsigDB.drug*

Each KEA3 candidate library was evaluated to see how well it recovers the ‘true’ kinase(s) in the query protein set from the benchmark datasets. ROC and PR curves were constructed from the scaled ranks of the ‘true’ kinases associated with the query set, composing the positive class, with the scaled rankings of the kinases not associated with the query composing the negative class (Figure 3). The STRING library performed the best for the *KinCREEDSupdn* and *DrugL1000updn* benchmarking datasets, but interestingly, its performance falls for the *PTMsigDB.drug* dataset. In general, the PPI libraries performed better than the KSI libraries. HIPPIE, prePPI, and mentha were the best-performing PPI libraries for *KinCREEDSupdn*; HIPPIE, mentha, and String.bind were the best performing PPI libraries for the *DrugL1000updn* dataset; and HIPPIE, mentha, and Cheng.PPI were the overall best performers for the *PTMsigDB.drug* dataset. The KSI libraries’ performance improved in the *PTMsigDB.drug* benchmarking dataset compared to the other two benchmarking datasets. This may be because the *PTMsigDB.drug* dataset is derived from a readout type that directly measures kinase activity. The top performing KSI libraries in this benchmark were Cheng.KSIN, PhosphoSitePlus, and PTMsigDB.

### Benchmarking the integrative methods

**Figure 4.**
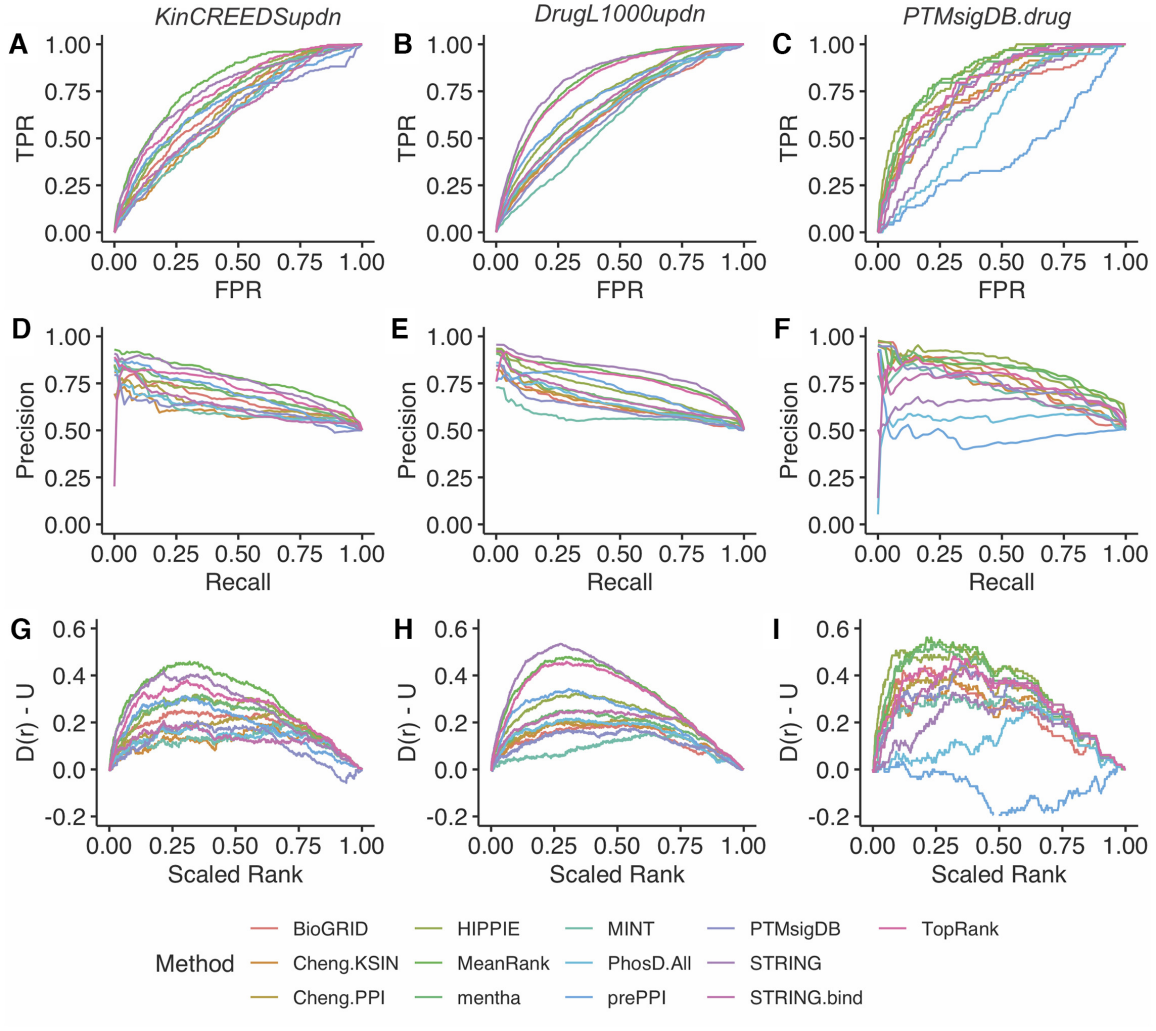
Performance of the final selected KEA3 libraries and integration methods in recovering the perturbed and drug-targeted kinase(s) from the three benchmarking datasets. (**A**–**C**) ROC curves for *KinCREEDSupdn*, *DrugL1000updn*, and *PTMsigDB.drug*, respectively; (**D**–**F**) Composite PR curves generated from 5,000 bootstrapped curves for *KinCREEDSupdn*, *DrugL1000updn*, and *PTMsigDB.drug*, respectively; (**G**–**I**) The deviation of the cumulative distribution from uniform of the scaled rankings of the ‘true’ kinases for *KinCREEDSupdn*, *DrugL1000updn* and *PTMsigDB.drug*, respectively.

**Figure 5.**
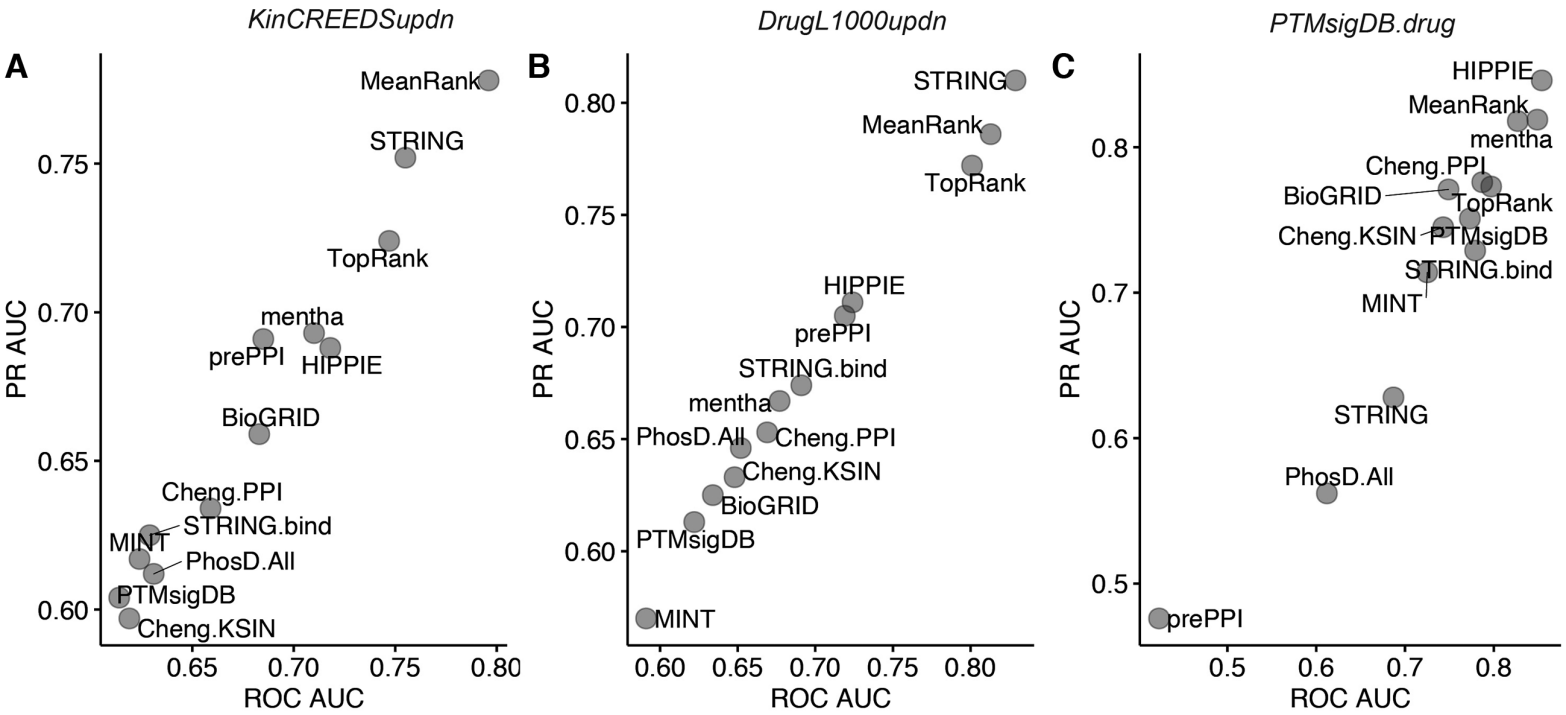
ROC AUC and mean PR AUC over 1,000 bootstrapped PR curves for the final selected KEA3 libraries and integration methods from three benchmarking datasets. (**A**) *KinCREEDSupdn*; (**B**) *DrugL1000updn;* (**C**) *PTMsigDB.drug*.

To construct the final KEA3 library set, we selected the 11 libraries with a ROC AUC and mean PR AUC in the top 50% of all libraries for at least two of the three benchmarks. Using the 11 libraries that passed this threshold, we assessed the predictive performance of two integration methods, MeanRank and TopRank, as previously described (23) for the three benchmarking datasets (Figures 4 and 5). MeanRank was the top-performing method for the *KinCREEDSupdn* dataset and was second-best to STRING for the *DrugL1000updn* dataset. MeanRank was also second to HIPPIE for the *PTMsigDB.drug* dataset. The TopRank integration method performed third, third and fifth for the *KinCREEDSupdn*, *DrugL1000updn* and *PTMsigDB.drug* datasets. While MeanRank was not the best performer in all three benchmarking datasets, it was the most consistent, as it is the only method to always be among the top two performers.

### Using the KEA3 web application

When users first navigate to the KEA3 homepage (https://maayanlab.cloud/kea3/), they are presented with an input form. To begin an analysis session, users would need to paste a list of proteins encoded as human or mouse gene symbols. Alternatively, users may also upload an existing text file containing the protein names, with one entry per line. KEA3 currently supports HGNC-approved gene symbols, and the webserver application will automatically tell users if there are any invalid or duplicate symbols in their input. Once the input has been submitted, the user may scroll down to view the kinase enrichment results. The ‘Integrated results’ tab is displayed by default, and shows the bar charts, tables, subnetwork and clustergrammer visualizations for the MeanRank and TopRank methods. These integrated results are shown on the first tab because they account for the results across all libraries, are less redundant, and performed well across the KEA3 benchmarks. Individual library results can be accessed using the other tabs.

The ‘Tables’ tab displays the kinase rankings for each of the KEA3 libraries, as determined by the Fisher's Exact Test *P*-value. Top-ranked kinases in all tables are those which have putative substrates that overlap the most with the input set. Users may sort tables by any of the columns simply by clicking on the column header or search for specific kinases using the search box above each table. Full results from each table may also be downloaded as a tab-separated (.tsv) file.

Visualizations are also provided for each of the kinase co-expression networks generated from the GTEx, TCGA and ARCHS4 expression data in the ‘Networks’ tab. Users can select any of the libraries for visualization using the drop-down menu. The top-ranked kinases are highlighted with their symbols shown. Users may additionally select to label kinases by either WGCNA modules or dataset-specific labels. All network visualizations can be downloaded as a scalable vector graphics (.svg) file or an image (.png). Kinase co-regulatory network visualizations can be found under the ‘Subnetworks’ tab and are dynamically generated from the top-ranked kinases in each library. An edge between two kinase nodes indicates an interaction supported by library evidence from either a KSI library (directed edge) or from a PPI library (undirected edge). Hovering over an edge will display the library evidence supporting the interaction. Each network can be downloaded as a scalable vector graphic (.svg) file or an raster image (.png). The ‘Bar Charts’ tab provides bar charts which show the -log(*P*-value) of the top-ranked kinases for each of the individual libraries. The ‘Clustergrammer’ tab provides an interactive clustergram of overlapping substrate targets between the input and the top library results, produced using the Clustergrammer application (68).

### The KEA3 Appyter

To provide users with the option to obtain KEA3 results as a downloadable Jupyter Notebook, we also developed the KEA3 Appyter. Appyters are standalone web-based applications that generate a Jupyter Notebook from a user input (69). The KEA3 Appyter takes as input a list of proteins, for example, differentially phosphorylated proteins, in the form of plain text or a text file. Using the KEA3 API, the Appyter queries the KEA3 server, and displays the results as a Jupyter Notebook. The notebook displays the results as an interactive bar chart and with tables of the top 10 kinases for integrated scores and all individual KEA3 libraries. This Jupyter Notebook can be saved, repurposed for different inputs, or used as part of other analysis pipelines and workflows. The KEA3 Appyter is available at: https://appyters.maayanlab.cloud/KEA3_Appyter/

### The SARS-CoV-2 kinase enrichment analysis case study

**Figure 6.**
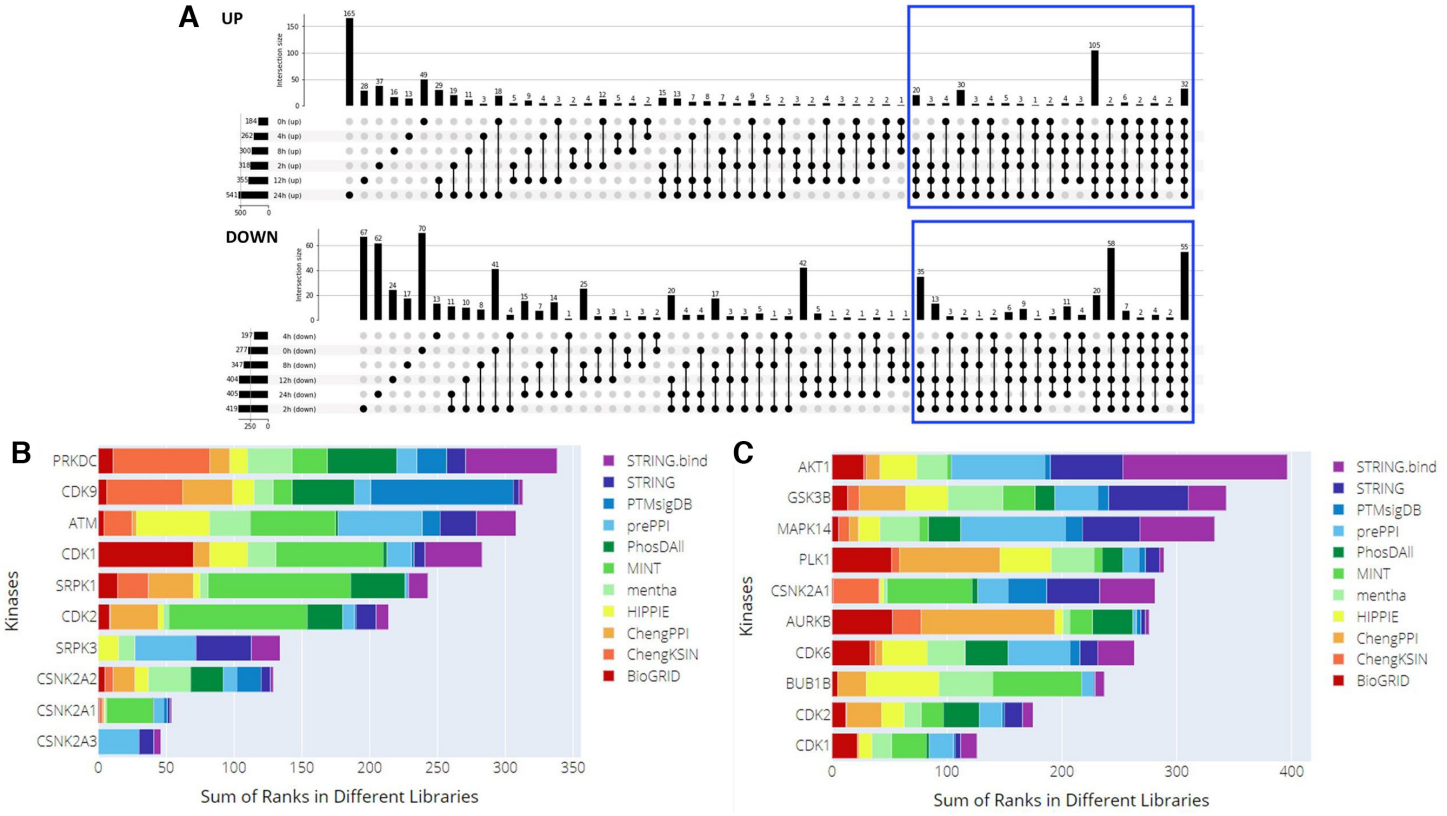
KEA3 analysis of the phosphoproteomics consensus sets from the SARS-CoV-2 study. (**A**) UpSet plot demonstrating the inclusion of consensus up- and down-regulated proteins used as input to KEA3. (**B**) MeanRank visualization from KEA3 for the up-phosphorylated proteins. **(C)** MeanRank visualization from KEA3 for the down-phosphorylated proteins.

Over the past year, the coronavirus disease 2019 (COVID-19) pandemic caused by the severe acute respiratory syndrome coronavirus 2 (SARS-CoV-2) virus has become a predominant focus of the scientific research community. Many research teams have altered their focus toward gaining a better understanding of the viral mechanisms underlying SARS-CoV-2 infection. Currently, there is still much unknown about SARS-CoV-2 infection and replication within human cells. In this case study, we attempt to demonstrate how kinase enrichment analysis using KEA3 can be applied to data compiled from a recent phosphoproteomics study to provide additional insight on some of those intracellular molecular mechanisms. The phosphoproteomics data were derived from a study of the phosphorylation changes induced by SARS-CoV-2 infection in Vero E6 cells (70). Up- and down-phosphorylated consensus protein sets were generated by filtering the data for phosphosites with log2(fold change) >1 and adjusted p-value <0.05 for each SARS-CoV-2 infection time point, extracting all proteins which were up-phosphorylated for at least four time points (Figure 6A), and removing duplicate entries. To identify potential upstream regulatory mechanisms responsible for the observed changes in protein phosphorylation upon SARS-CoV-2 infection, or involved in viral-host protein interactions, we performed kinase enrichment analysis on each of the consensus sets created above using KEA3 (Figure 6B and C).

The top ranked most enriched kinases for the up-phosphorylated proteins show three members of the casein kinase family. Casein kinases are serine/threonine kinases that participate in many cell-signaling pathways, including DNA repair (71). It was recently shown that CSNK2A2 directly interacts with SARS-CoV-2 N protein (72). Hence, it is possible that viral evading strategies are mediated by altering cell-signaling regulated by CSNK2A2. The top ten enriched kinases for the up-phosphorylated proteins also include SRPK1 and SRPK3. SRPK1 is highly expressed in most tissues and mostly associated with DNA and RNA processing (73), while SRPK3 is involved more specifically in muscle related functions (74) and as such could be linked to cardiac complications observed in some COVID-19 patients (75). Another interesting protein kinase that is found in the top-ranked up-phosphorylated proteins is CDK9. In previous studies, activated CDK9 has been demonstrated to play a role in regulating innate immune responses (76).

The top kinases enriched for the phosphoproteomics consensus down set are CDK1 and CDK2, suggesting down-regulation of the cell cycle. This is a common cellular immune response upon viral or bacterial infection of Vero cells. Other top kinases include p38, GSK3B, and AKT1 which are known as the downstream kinases for several interleukin signals. In addition, AURKB is a cell cycle kinase that plays a significant role in chromosome segregation during mitosis (77), while GSK3B is a serine-threonine kinase and part of the glycogen synthase kinase-3 family that has been associated with viral genome replication in COVID-19 (78). While further study is needed to elucidate the specific impact of these kinases in SARS-CoV-2 and other viral infections, this case study illustrates the usefulness and applicability of KEA3 to current and future phosphoproteomics studies. Using the KEA3 approach, kinase inhibitors could be designed to mitigate the effect of SARS-CoV-2 on cells, although this needs to be done carefully because some identified kinases are part of innate immune response pathways, while others are altered by the virus to evade such immune responses.

## SUMMARY

Phosphoproteomics efforts have detected tens of thousands of phosphorylation sites in cellular proteins. However, in most cases, the kinases that are responsible for these post-translational modifications are unknown. For instance, less than 5% of the phosphorylation sites in PhosphoSitePlus are annotated with kinases (52,53). To develop KEA3 we combined directly measured KSIs, withPPIs and co-expression data sources to predict upstream kinases given lists of differentially phosphorylated proteins. PPI detection methods do not uncover the directionality or effect of the interaction between two proteins; however, we used these datasets as a proxy for KSIs. In this same vein, we also included kinase co-expression libraries with the notion that members of pathways tend to be co-expressed (79). While ultimately the co-expression libraries did not show a strong enough signal in our benchmarks to pass the threshold for inclusion in the final set of 11 libraries used within the KEA3 web-server application, these are made available for download from the KEA3 website.

The approach we used to assess interlibrary predictability (Figure 2) simultaneously evaluates: (i) the concordance of sets associated with the same kinases within the library pair under consideration and (ii) how well a given library can distinguish between kinases. The libraries derived from PPI sources show high inter-library predictability, which is unsurprising given the substantial redundancy among many sources (29,30,34,41). The AUCs for PPI-KSI library pairs indicate that, while PPIs may be a less direct source for kinase substrates than directly measured KSIs, PPIs are useful in identifying the correct upstream kinases.

We used three independent benchmarking datasets to evaluate the predictive performance of the KEA3 candidate libraries. The *KinCREEDSupdn* and *DrugL1000updn* datasets are derived from gene expression signatures. They rely on the assumption that when a kinase is perturbed experimentally or is the target of a small molecule, the transcripts encoding the kinase's substrates will also be measurably perturbed as a downstream effect. The signal in ROC, PR, and bridge plots of the libraries derived from KSI sources tested on these benchmarking datasets supports this hypothesis. The *PTMsigDB.drug* benchmarking dataset more directly tests the predictive performance of KEA3 by querying the libraries with drug perturbation phosphoproteomics signatures, with the underlying assumption being that the substrates of the small molecule-affected kinase(s) will be differentially phosphorylated. However, experiments measuring global changes in phosphorylation following perturbation are few, and the *PTMsigDB.drug* benchmarking set is small. Taken together, however, the three benchmarking sets indicate the KEA3 candidate libraries' comparative performance, as well as the performance of the two integrative methods, MeanRank and TopRank. MeanRank performed consistently well across the benchmarking datasets. The two top-performing libraries, STRING and HIPPIE, displayed variable performance depending on the benchmark query type. We would therefore recommend that users rely most heavily on the integrated MeanRank method. Finally, by reprocessing data from a recent SARS-CoV-2 phosphoproteomics study (70), we demonstrate how KEA3 can complement the analysis of differential mass-spectrometry phosphoproteomics studies. Our results are consistent with the authors of the original study, but also add clarity and confirmation about the key kinases involved.

It should be noted that kinase activity may not change, even if the modification level of their substrates increased or decreased. This is because the kinases and their substrates function in a complex environment that involves other interacting proteins. For example, the increase or decrease in the phosphorylation level of substrate proteins for a specific kinase might be attributed to changes in their localization, interactions with other partners, or due to competition with phosphatases that may also increase or decrease in quantity and/or activity.

Overall, KEA3 can be a useful tool for biologists to generate hypotheses from gene expression and phosphoproteomic profiling experiments. We note that KEA3 relies heavily on libraries with knowledge curated from the literature or high-throughput experiments on well-characterized kinases. Literature-based PPI and KSI interactions suffer from research focus biases where well-studied proteins are overrepresented (80). Expanding KEA3 libraries to incorporate global studies of kinase state and lesser-studied kinases (11) is the subject of future work. Future work will also include connecting top predicted kinases to the known small molecules that target them.

## References

[1] Burnett G. , Kennedy E.P. The enzymatic phosphorylation of proteins. J. Biol. Chem. 1954; 211:969–980. 13221602

[2] Walsh D.A. , Perkins J.P., Krebs E.G. An adenosine 3′,5′-monophosphate-dependant protein kinase from rabbit skeletal muscle. J. Biol. Chem. 1968; 243:3763–3765. 4298072

[3] Ubersax J.A. , Ferrell J.E. Jr Mechanisms of specificity in protein phosphorylation. Nat. Rev. Mol. Cell Biol. 2007; 8:530–541. 17585314 10.1038/nrm2203

[4] Sharma K. , D'Souza R.C.J., Tyanova S., Schaab C., Wiśniewski J.R., Cox J., Mann M. Ultradeep human phosphoproteome reveals a distinct regulatory nature of Tyr and Ser/Thr-based signaling. Cell Rep. 2014; 8:1583–1594. 25159151 10.1016/j.celrep.2014.07.036

[5] Manning G. , Whyte D.B., Martinez R., Hunter T., Sudarsanam S. The protein kinase complement of the human genome. Science. 2002; 298:1912–1934. 12471243 10.1126/science.1075762

[6] Rowley J.D. Letter: A new consistent chromosomal abnormality in chronic myelogenous leukaemia identified by quinacrine fluorescence and Giemsa staining. Nature. 1973; 243:290–293. 4126434 10.1038/243290a0

[7] Collins S.J. , Groudine M.T. Rearrangement and amplification of c-abl sequences in the human chronic myelogenous leukemia cell line K-562. Proc. Natl. Acad. Sci. U.S.A. 1983; 80:4813–4817. 6308652 10.1073/pnas.80.15.4813 PMC384135

[8] George S. , Rochford J.J., Wolfrum C., Gray S.L., Schinner S., Wilson J.C., Soos M.A., Murgatroyd P.R., Williams R.M., Acerini C.L. et al. A family with severe insulin resistance and diabetes due to a mutation in AKT2. Science. 2004; 304:1325–1328. 15166380 10.1126/science.1096706 PMC2258004

[9] Alsina-Sanchís E. , García-Ibáñez Y., Figueiredo A.M., Riera-Domingo C., Figueras A., Matias-Guiu X., Casanovas O., Botella L.M., Pujana M.A., Riera-Mestre A. et al. ALK1 loss results in vascular hyperplasia in mice and humans through PI3K activation. Arterioscler. Thromb. Vasc. Biol. 2018; 38:1216–1229. 29449337 10.1161/ATVBAHA.118.310760

[10] White M.J. , Phillip Morris C., Lawford B.R., Young RMcD Behavioral phenotypes of impulsivity related to the ANKK1 gene are independent of an acute stressor. Behav Brain Funct. 2008; 4:54. 19025655 10.1186/1744-9081-4-54 PMC2607297

[11] Rodgers G. , Austin C., Anderson J., Pawlyk A., Colvis C., Margolis R., Baker J. Glimmers in illuminating the druggable genome. Nat. Rev. Drug Discov. 2018; 17:301–302. 29348682 10.1038/nrd.2017.252 PMC6309548

[12] Ferguson F.M. , Gray N.S. Kinase inhibitors: the road ahead. Nat. Rev. Drug Discov. 2018; 17:353–377. 29545548 10.1038/nrd.2018.21

[13] Mann M. , Ong SEn, Grønborg M., Steen H., Jensen O.N., Pandey A. Analysis of protein phosphorylation using mass spectrometry: deciphering the phosphoproteome. Trends Biotechnol. 2002; 20:261–268. 12007495 10.1016/s0167-7799(02)01944-3

[14] Casado P. , Rodriguez-Prados J.-.C., Cosulich S.C., Guichard S., Vanhaesebroeck B., Joel S., Cutillas P.R. Kinase-substrate enrichment analysis provides insights into the heterogeneity of signaling pathway activation in leukemia cells. Sci. Signal. 2013; 6:rs6. 23532336 10.1126/scisignal.2003573

[15] Chen E.Y. , Xu H., Gordonov S., Lim M.P., Perkins M.H., Ma’ayan A. Expression2Kinases: mRNA profiling linked to multiple upstream regulatory layers. Bioinformatics. 2012; 28:105–111. 22080467 10.1093/bioinformatics/btr625 PMC3244772

[16] Clarke D.J.B. , Kuleshov M.V., Schilder B.M., Torre D., Duffy M.E., Keenan A.B., Lachmann A., Feldmann A.S., Gundersen G.W., Silverstein M.C. et al. eXpression2Kinases (X2K) Web: linking expression signatures to upstream cell signaling networks. Nucleic. Acids. Res. 2018; 46:W171–W179. 29800326 10.1093/nar/gky458 PMC6030863

[17] Krug K. , Mertins P., Zhang B., Hornbeck P., Raju R., Ahmad R., Szucs M., Mundt F., Forestier D., Jane-Valbuena J. et al. A curated resource for phosphosite-specific signature analysis. Mol. Cell. Proteomics. 2019; 18:576–593. 10.1074/mcp.TIR118.000943 PMC6398202 30563849

[18] Mischnik M. , Sacco F., Cox J., Schneider H.-.C., Schäfer M., Hendlich M., Crowther D., Mann M., Klabunde T. IKAP: A heuristic framework for inference of kinase activities from phosphoproteomics data. Bioinformatics. 2016; 32:424–431. 26628587 10.1093/bioinformatics/btv699

[19] Yang P. , Patrick E., Humphrey S.J., Ghazanfar S., James D.E., Jothi R., Yang J.Y.H. KinasePA: Phosphoproteomics data annotation using hypothesis driven kinase perturbation analysis. Proteomics. 2016; 16:1868–1871. 27145998 10.1002/pmic.201600068 PMC5027648

[20] Wiredja D.D. , Koyutürk M., Chance M.R. The KSEA App: a web-based tool for kinase activity inference from quantitative phosphoproteomics. Bioinformatics. 2017; 33:3489–3491. 28655153 10.1093/bioinformatics/btx415 PMC5860163

[21] Lachmann A. , Xu H., Krishnan J., Berger S.I., Mazloom A.R., Ma’ayan A. ChEA: transcription factor regulation inferred from integrating genome-wide ChIP-X experiments. Bioinformatics. 2010; 26:2438–2444. 20709693 10.1093/bioinformatics/btq466 PMC2944209

[22] Kou Y. , Chen EY., Clark NR., Duan Q., Tan CM., Ma‘ayan A. ChEA2: Gene-Set Libraries from ChIP-X Experiments to Decode the Transcription Regulome. 2013; Berlin, Heidelberg Springer.

[23] Keenan A.B. , Torre D., Lachmann A., Leong A.K., Wojciechowicz M.L., Utti V., Jagodnik K.M., Kropiwnicki E., Wang Z., Ma’ayan A. ChEA3: transcription factor enrichment analysis by orthogonal omics integration. Nucleic. Acids. Res. 2019; 47:W212–W224. 31114921 10.1093/nar/gkz446 PMC6602523

[24] Berger S.I. , Posner J.M., Ma’ayan A. Genes2Networks: connecting lists of gene symbols using mammalian protein interactions databases. BMC Bioinformatics. 2007; 8:372. 17916244 10.1186/1471-2105-8-372 PMC2082048

[25] Lachmann A. , Ma’ayan A. KEA: kinase enrichment analysis. Bioinformatics. 2009; 25:684–686. 19176546 10.1093/bioinformatics/btp026 PMC2647829

[26] Braschi B. , Denny P., Gray K., Jones T., Seal R., Tweedie S., Yates B., Bruford E. Genenames.org: the HGNC and VGNC resources in 2019. Nucleic. Acids. Res. 2019; 47:D786–D792. 30304474 10.1093/nar/gky930 PMC6324057

[27] Miranda-Saavedra D. , Barton G.J. Classification and functional annotation of eukaryotic protein kinases. Proteins. 2007; 68:893–914. 17557329 10.1002/prot.21444

[28] Szklarczyk D. , Gable A.L., Lyon D., Junge A., Wyder S., Huerta-Cepas J., Simonovic M., Doncheva N.T., Morris J.H., Bork P. et al. STRING v11: protein–protein association networks with increased coverage, supporting functional discovery in genome-wide experimental datasets. Nucleic Acids Res. 2018; 47:D607–D613. 10.1093/nar/gky1131 PMC6323986 30476243

[29] Oughtred R. , Stark C., Breitkreutz B.-.J., Rust J., Boucher L., Chang C., Kolas N., O’Donnell L., Leung G., McAdam R. et al. The BioGRID interaction database: 2019 update. Nucleic. Acids. Res. 2019; 47:D529–D541. 30476227 10.1093/nar/gky1079 PMC6324058

[30] Calderone A. , Castagnoli L., Cesareni G. entha: a resource for browsing integrated protein-interaction networks. Nat. Methods. 2013; 10:690–691. 23900247 10.1038/nmeth.2561

[31] Drew K. , Lee C., Huizar R.L., Tu F., Borgeson B., McWhite C.D., Ma Y., Wallingford J.B., Marcotte E.M. Integration of over 9,000 mass spectrometry experiments builds a global map of human protein complexes. Mol. Syst. Biol. 2017; 13:932. 28596423 10.15252/msb.20167490 PMC5488662

[32] Zhang Q.C. , Petrey D., Ignacio Garzón J., Deng L., Honig B. PrePPI: a structure-informed database of protein-protein interactions. Nucleic. Acids. Res. 2013; 41:D828–D833. 23193263 10.1093/nar/gks1231 PMC3531098

[33] Zhang Q.C. , Petrey D., Deng L., Qiang Li, Shi Yu, Thu C.A., Bisikirska B., Lefebvre C., Accili D., Hunter T. et al. Structure-based prediction of protein-protein interactions on a genome-wide scale. Nature. 2012; 490:556–560. 23023127 10.1038/nature11503 PMC3482288

[34] Licata L. , Briganti L., Peluso D., Perfetto L., Iannuccelli M., Galeota E., Sacco F., Palma A., Nardozza A.P., Santonico E. et al. MINT, the molecular interaction database: 2012 update. Nucleic. Acids. Res. 2012; 40:D857–D861. 22096227 10.1093/nar/gkr930 PMC3244991

[35] Licata L. , Orchard S. The MIntAct project and molecular interaction databases. Methods Mol. Biol. 2016; 1415:55–69. 27115627 10.1007/978-1-4939-3572-7_3

[36] Alanis-Lobato G. , Andrade-Navarro M.A., Schaefer M.H. HIPPIE v2.0: enhancing meaningfulness and reliability of protein-protein interaction networks. Nucleic. Acids. Res. 2017; 45:D408–D414. 27794551 10.1093/nar/gkw985 PMC5210659

[37] McDowall M.D. , Scott M.S., Barton G.J. PIPs: human protein-protein interaction prediction database. Nucleic. Acids. Res. 2009; 37:D651–D656. 18988626 10.1093/nar/gkn870 PMC2686497

[38] Scott M.S. , Barton G.J. Probabilistic prediction and ranking of human protein-protein interactions. BMC Bioinformatics. 2007; 8:239. 17615067 10.1186/1471-2105-8-239 PMC1939716

[39] Murakami Y. , Mizuguchi K. PSOPIA: toward more reliable protein-protein interaction prediction from sequence information. 2017 International Conference on Intelligent Informatics and Biomedical Sciences. 2017; ICIIBMS.

[40] Fabregat A. , Sidiropoulos K., Garapati P., Gillespie M., Hausmann K., Haw R., Jassal B., Jupe S., Korninger F., McKay S. et al. The reactome pathway knowledgebase. Nucleic. Acids. Res. 2016; 44:D481–D487. 26656494 10.1093/nar/gkv1351 PMC4702931

[41] Cheng F. , Jia P., Wang Q., Zhao Z. Quantitative network mapping of the human kinome interactome reveals new clues for rational kinase inhibitor discovery and individualized cancer therapy. Oncotarget. 2014; 5:3697–3710. 25003367 10.18632/oncotarget.1984 PMC4116514

[42] Orchard S. , Ammari M., Aranda B., Breuza L., Briganti L., Broackes-Carter F., Campbell N.H., Chavali G., Chen C., del-Toro N. et al. The MIntAct project–IntAct as a common curation platform for 11 molecular interaction databases. Nucleic. Acids. Res. 2014; 42:D358–D363. 24234451 10.1093/nar/gkt1115 PMC3965093

[43] Clerc O. , Deniaud M., Vallet S.D., Naba A., Rivet A., Perez S., Thierry-Mieg N., Ricard-Blum S. MatrixDB: integration of new data with a focus on glycosaminoglycan interactions. Nucleic Acids Res. 2019; 47:D376–D381. 30371822 10.1093/nar/gky1035 PMC6324007

[44] Salwinski L. , Miller C.S., Smith A.J., Pettit F.K., Bowie J.U., Eisenberg D. The database of interacting proteins: 2004 update. Nucleic Acids Res. 2004; 32:D449–D451. 14681454 10.1093/nar/gkh086 PMC308820

[45] Keshava Prasad T.S. , Goel R., Kandasamy K., Keerthikumar S., Kumar S., Mathivanan S., Telikicherla D., Raju R., Shafreen B., Venugopal A. et al. Human Protein Reference Database–2009 update. Nucleic Acids Res. 2009; 37:D767–D772. 18988627 10.1093/nar/gkn892 PMC2686490

[46] Bader G.D. , Betel D., Hogue C.W. BIND: the Biomolecular Interaction Network Database. Nucleic. Acids. Res. 2003; 31:248–250. 12519993 10.1093/nar/gkg056 PMC165503

[47] Pagel P. , Kovac S., Oesterheld M., Brauner B., Dunger-Kaltenbach I., Frishman G., Montrone C., Mark P., Stümpflen V., Mewes H.-.W. et al. The MIPS mammalian protein-protein interaction database. Bioinformatics. 2005; 21:832–834. 15531608 10.1093/bioinformatics/bti115

[48] Güldener U. , Münsterkötter M., Oesterheld M., Pagel P., Ruepp A., Mewes H.-.W., Stümpflen V. MPact: the MIPS protein interaction resource on yeast. Nucleic Acids Res. 2006; 34:D436–D441. 16381906 10.1093/nar/gkj003 PMC1347366

[49] Fabregat A. , Sidiropoulos K., Garapati P., Gillespie M., Hausmann K., Haw R., Jassal B., Jupe S., Korninger F., McKay S., Matthews L. et al. The reactome pathway knowledgebase. Nucleic Acids Res. 2018; 46:D649–D655. 29145629 10.1093/nar/gkx1132 PMC5753187

[50] Hu J. , Rho H.-.S., Newman R.H., Zhang J., Zhu H., Qian J. PhosphoNetworks: a database for human phosphorylation networks. Bioinformatics. 2014; 30:141–142. 24227675 10.1093/bioinformatics/btt627 PMC3866559

[51] Dinkel H. , Chica C., Via A., Gould C.M., Jensen L.J., Gibson T.J., Diella F. Phospho.ELM: a database of phosphorylation sites–update 2011. Nucleic Acids Res. 2011; 39:D261–D267. 21062810 10.1093/nar/gkq1104 PMC3013696

[52] Hornbeck P.V. , Zhang B., Murray B., Kornhauser J.M., Latham V., Skrzypek E. PhosphoSitePlus, 2014: mutations, PTMs and recalibrations. Nucleic Acids Res. 2015; 43:D512–D520. 25514926 10.1093/nar/gku1267 PMC4383998

[53] Qin G.M. , Li R.Y., Zhao X.M. PhosD: inferring kinase-substrate interactions based on protein domains. Bioinformatics. 2017; 33:1197–1204. 28031187 10.1093/bioinformatics/btw792

[54] Hu J. , Rho H.-.S., Newman R., Hwang W., Neiswinger J., Zhu H., Zhang J., Qian J. Global analysis of phosphorylation networks in humans. Biochim. Biophys. Acta. 2014; 1844:224–231. 23524292 10.1016/j.bbapap.2013.03.009 PMC3815481

[55] Newman R.H. , Hu J., Rho H.-.S., Xie Z., Woodard C., Neiswinger J., Cooper C., Shirley M., Clark H.M., Hu S. et al. Construction of human activity-based phosphorylation networks. Mol. Syst. Biol. 2013; 9:655. 23549483 10.1038/msb.2013.12 PMC3658267

[56] Lachmann A. , Torre D., Keenan A.B., Jagodnik K.M., Lee H.J., Wang L., Silverstein M.C., Ma’ayan A. Massive mining of publicly available RNA-seq data from human and mouse. Nat. Commun. 2018; 9:1366. 29636450 10.1038/s41467-018-03751-6 PMC5893633

[57] Clark N.R. , Hu K.S., Feldmann A.S., Kou Y., Chen E.Y., Duan Q., Ma’ayan A. The characteristic direction: a geometrical approach to identify differentially expressed genes. BMC Bioinformatics. 2014; 15:79. 24650281 10.1186/1471-2105-15-79 PMC4000056

[58] Wang Z. , Monteiro C.D., Jagodnik K.M., Fernandez N.F., Gundersen G.W., Rouillard A.D., Jenkins S.L., Feldmann A.S., Hu K.S., McDermott M.G. et al. Extraction and analysis of signatures from the Gene Expression Omnibus by the crowd. Nat. Commun. 2016; 7:12846. 27667448 10.1038/ncomms12846 PMC5052684

[59] Wang Z. , Lachmann A., Keenan A.B., Ma’ayan A. L1000FWD: fireworks visualization of drug-induced transcriptomic signatures. Bioinformatics. 2018; 34:2150–2152. 29420694 10.1093/bioinformatics/bty060 PMC6454499

[60] Garcia-Alonso L. , Holland C.H., Ibrahim M.M., Turei D., Saez-Rodriguez J. Benchmark and integration of resources for the estimation of human transcription factor activities. Genome Res. 2019; 29:1363–1375. 31340985 10.1101/gr.240663.118 PMC6673718

[61] Brittain J. , Darwin I.F. Tomcat: the definitive guide. 2007; 2nd edn O’Reilly.

[62] Mobirise 2018; https://mobirise.com/.

[63] Merkel D. Docker: lightweight Linux containers for consistent development and deployment. Linux J. 2014; 2014:Article 2.

[64] Langfelder P. , Horvath S. WGCNA: an R package for weighted correlation network analysis. BMC Bioinformatics. 2008; 9:559. 19114008 10.1186/1471-2105-9-559 PMC2631488

[65] The Genotype-Tissue Expression (GTEx) project. Nat. Genet. 2013; 45:580–585. 23715323 10.1038/ng.2653 PMC4010069

[66] Shannon P. , Markiel A., Ozier O., Baliga N.S., Wang J.T., Ramage D., Amin N., Schwikowski B., Ideker T. Cytoscape: a software environment for integrated models of biomolecular interaction networks. Genome Res. 2003; 13:2498–2504. 14597658 10.1101/gr.1239303 PMC403769

[67] Bostock M. , Ogievetsky V., Heer J. D³Data-driven documents. IEEE Trans. Vis. Comput. Graph. 2011; 17:2301–2309. 22034350 10.1109/TVCG.2011.185

[68] Fernandez N.F. , Gundersen G.W., Rahman A., Grimes M.L., Rikova K., Hornbeck P., Ma’ayan A. Clustergrammer, a web-based heatmap visualization and analysis tool for high-dimensional biological data. Scientific Data. 2017; 4:170151. 28994825 10.1038/sdata.2017.151 PMC5634325

[69] Clarke D.J.B. , Jeon M., Stein DJ., Moiseyev N., Kropiwnicki E., Dai C., Xie Z., Wojciechowicz M., Litz S., Hom J. et al. Appyters: turning Jupyter Notebooks into data-driven web apps. Patterns. 2021; 2:100213. 33748796 10.1016/j.patter.2021.100213 PMC7961182

[70] Bouhaddou M. , Memon D., Meyer B., White K.M., Rezelj V.V., Marrero M.C., Polacco B.J., Melnyk J.E., Ulferts S., Kaake R.M. et al. The Global Phosphorylation Landscape of SARS-CoV-2 Infection. Cell. 2020; 182:685–712. 32645325 10.1016/j.cell.2020.06.034 PMC7321036

[71] Loizou J.I. , El-Khamisy S.F., Zlatanou A., Moore D.J., Chan D.W., Qin J., Sarno S., Meggio F., Pinna L.A., Caldecott K.W. The protein kinase CK2 facilitates repair of chromosomal DNA single-strand breaks. Cell. 2004; 117:17–28. 15066279 10.1016/s0092-8674(04)00206-5

[72] Gordon D.E. , Jang G.M., Bouhaddou M., Xu J., Obernier K., White K.M., O’Meara M.J., Rezelj V.V., Guo J.Z., Swaney D.L. et al. A SARS-CoV-2-human protein-protein interaction map reveals drug targets and potential drug-repurposing. 2020; bioRxiv doi:23 March 2020, preprint: not peer reviewed 10.1101/2020.03.22.002386. PMC7431030 32353859

[73] Aubol B.E. , Wu G., Keshwani M.M., Movassat M., Fattet L., Hertel K.J., Fu X.-.D., Adams J.A. Release of SR proteins from CLK1 by SRPK1: a symbiotic kinase system for phosphorylation control of pre-mRNA splicing. Mol. Cell. 2016; 63:218–228. 27397683 10.1016/j.molcel.2016.05.034 PMC4941815

[74] Nakagawa O. , Arnold M., Nakagawa M., Hamada H., Shelton J.M., Kusano H., Harris T.M., Childs G., Campbell K.P., Richardson J.A. et al. Centronuclear myopathy in mice lacking a novel muscle-specific protein kinase transcriptionally regulated by MEF2. Genes Dev. 2005; 19:2066–2077. 16140986 10.1101/gad.1338705 PMC1199576

[75] Samidurai A. , Das A. Cardiovascular complications associated with COVID-19 and potential therapeutic strategies. Int. J. Mol. Sci. 2020; 21:6790. 10.3390/ijms21186790 PMC7554795 32947927

[76] Tian B. , Zhao Y., Kalita M., Edeh C.B., Paessler S., Casola A., Teng M.N., Garofalo R.P., Brasier A.R. CDK9-dependent transcriptional elongation in the innate interferon-stimulated gene response to respiratory syncytial virus infection in airway epithelial cells. J. Virol. 2013; 87:7075–7092. 23596302 10.1128/JVI.03399-12 PMC3676079

[77] Gully C.P. , Velazquez-Torres G., Shin Ji-H, Fuentes-Mattei E., Wang E., Carlock C., Chen J., Rothenberg D., Adams HP., Choi HHo, Guma S. et al. Aurora B kinase phosphorylates and instigates degradation of p53. Proc. Natl. Acad. Sci. U.S.A. 2012; 109:E1513–E1522. 22611192 10.1073/pnas.1110287109 PMC3386093

[78] Rana A.K. , Rahmatkarab S.N., Kumarab A., Singhab D. Glycogen synthase kinase-3: a putative target to combat severe acute respiratory syndrome coronavirus 2 (SARS-CoV-2) pandemic. Cytokine Growth Factor Rev. 2020; 58:92–101. 32948440 10.1016/j.cytogfr.2020.08.002 PMC7446622

[79] Pita-Juárez Y. , Altschuler G., Kariotis S., Wei W., Koler K., Green C., Tanzi RE., Hide W. The pathway coexpression network: revealing pathway relationships. PLoS Comput. Biol. 2018; 14:e1006042. 29554099 10.1371/journal.pcbi.1006042 PMC5875878

[80] Wang Z. , Clark N.R., Ma’ayan A. Ma’ayan, Dynamics of the discovery process of protein-protein interactions from low content studies. BMC Syst. Biol. 2015; 9:26. 26048415 10.1186/s12918-015-0173-z PMC4456804

